# Clinical outcomes in Fabry patients switching to agalsidase beta for renal ineffectiveness of the primary Fabry therapy: a single-centre analysis

**DOI:** 10.1093/ckj/sfaf318

**Published:** 2025-10-15

**Authors:** Eleonora Riccio, Ivana Capuano, Pasquale Buonanno, Guido Iaccarino, Calogero Lino Cirami, Renzo Mignani, Federico Pieruzzi, Antonio Pisani

**Affiliations:** Department of Public Health, Chair of Nephrology, University Federico II of Naples, Naples, Italy; Department of Public Health, Chair of Nephrology, University Federico II of Naples, Naples, Italy; Department of Neurosciences, Reproductive and Odontostomatological Sciences, University of Naples Federico II, Naples, Italy; Department of Clinical Medicine and Surgery, Federico II University, Naples, Italy; Nephrology Dialysis Transplant Unit, Careggi Hospital, Florence, Italy; Nephrology, Dialysis and Transplantation, IRCCS S. Orsola University Hospital, University of Bologna, Bologna, Italy; Nephrology, Fondazione IRCCS San Gerardo dei Tintori, Monza, Italy; School of Medicine and Surgery, University of Milano-Bicocca, Milan, Italy; Department of Public Health, Chair of Nephrology, University Federico II of Naples, Naples, Italy

**Keywords:** eGFR slope, enzyme replacement therapy, Fabry disease, renal effectiveness, switch

## Abstract

**Background:**

A treatment for classic Fabry disease (FD) should be defined effective on renal function when it maintains a decline of estimated glomerular filtration rate (eGFR) <1 ml/min/1.73 m^2^/year, and not effective when the eGFR loss remains ≥3 ml/min/1.73 m^2/^year. Therefore, considering the evidence of dose-dependent efficacy of the enzyme replacement therapy (ERT) and the data reporting the disease progression after switching form agalsidase beta to migalastat, a switch to higher doses of ERT should be considered in adult Fabry patients who have an eGFR of 45–90 ml/min/1.73 m^2^, with an eGFR slope ≥3 ml/min/1.73 m^2^/year. This study aimed to assess the effects of switching to agalsidase beta for renal ineffectiveness of the primary Fabry therapy.

**Methods:**

Data retrospectively taken during the pre-switch period were compared with data prospectively collected during the post-switch period. The primary endpoint was the effect on eGFR slope. Secondary endpoints were: changes in clinical events, 24-hour proteinuria, cardiac and neurologic parameters, FD-related symptoms, lyso-Gb3 plasma concentrations, and adverse events.

**Results:**

In total, 11 patients (nine males and two females) switched to agalsidase beta from 55.3 ± 31.2 months of primary therapy with agalsidase alfa (eight patients) or migalastat (three patients), were evaluated for a follow-up period of 24 months. After the switch, a significant reduction of the eGFR slope was observed (−4.61 vs −0.45 ml/min/1.73 m^2^/year, respectively in pre- and post-switch period; *P*_post__/pre_ < .005). After the switch, plasma lyso-Gb3 levels progressively reduced, and the reduction reached the significance vs baseline at T2 (*P* < .05). Most of FD-related symptoms ameliorated during the primary Fabry therapy and remained stable after the switch. All other parameters were stable over time.

**Conclusions:**

Treatment switch from agalsidase alfa or migalastat to agalsidase beta can attenuate eGFR decline and enhance lyso-Gb3 reduction, confirming the dose-dependent effect of agalsidase beta to further slow down FD progression.

KEY LEARNING POINTS **What was known:**

- Fabry disease (FD) leads to progressive nephropathy, and renal decline persists in many patients despite enzyme replacement therapy (ERT) or migalastat.
- Clinical guidelines suggest switching therapy when individualized renal goals are not achieved.

**This study adds:**

- Switching from agalsidase alfa or migalastat to agalsidase beta significantly attenuated eGFR decline in patients with ineffective primary therapy.
- Plasma lyso-Gb3 levels further decreased after switching, supporting the dose-dependent efficacy of agalsidase beta.
- Clinical symptoms, proteinuria, and cardiac/neurological outcomes remained stable, confirming safety and tolerability.

**Potential impact:**

- Agalsidase beta may be preferred in Fabry patients showing renal ineffectiveness under agalsidase alfa or migalastat.
- Therapy switch could help stabilize kidney function and improve biomarker control in real-world clinical practice.
- Findings reinforce the importance of individualized therapy optimization in Fabry disease management.

## INTRODUCTION

Renal involvement is one of the main features of Fabry disease (FD), due to the globotriaosylceramide (Gb3) deposition in all types of renal cell, and is characterized by a progressive nephropathy leading to kidney failure from the third to the fifth decade of life in untreated patients with classical mutations [1–4].

The availability of specific Fabry therapies modified the natural history of FD [5]. Enzyme replacement therapy (ERT) remains a cornerstone of FD management. Currently, three ERT formulations are available for Fabry treatment: agalsidase alfa, agalsidase beta, and the recently approved pegunigalsidase alfa. In particular, agalsidase alfa and beta [6, 7] have been widely used for years and have been shown to be effective on the progression of Fabry nephropathy; however, recent studies showed a dose-dependent effect of agalsidase on Gb3 clearance from cells [8, 9], and the highest doses have been reported to slow the progression of nephropathy [10, 11]. Moreover, treatment switch from agalsidase alfa to agalsidase beta has been shown to attenuate estimated glomerular filtration rate (eGFR) decline [11–13], which confirms the dose-dependent effect including a slowdown in renal disease progression, with agalsidase alfa and agalsidase beta being the two widely used forms.

Another clinically available approach is the oral chaperone migalastat [14]. To date, evidence in the literature has confirmed the efficacy and tolerability of migalastat in FD patients with amenable mutations [14], while the effects on long-term renal outcomes is not well defined: in Phase III studies [14, 15] and in the analysis of patients enrolled in the FollowME Pathfinders registry [16] stable eGFR values were reported for both naïve and ERT-experienced patients who switched to migalastat, while ‘real-world’ data revealed a more pronounced loss of eGFR in patients treated with the oral chaperone [17–19].

On the basis of data in the literature about eGFR decline in treated and untreated Fabry patients [13, 20–22], and on the evidence supporting the role of eGFR slope as a surrogate end point for chronic kidney disease (CKD) progression [23–26], it has been recently suggested that a therapy for classic FD should be defined effective on renal function when eGFR decline is <1 ml/min/1.73 m^2^ per year, and not effective when the eGFR loss remains ≥3 ml/min/1.73 m^2^ per year (≥2.5 ml/min/1.73 m^2^ per year in females) [27].

Moreover, practical clinical recommendations and guidance for Fabry patients suggested that treatment switch may be appropriate if individualized therapeutic goals are not achieved [28].

Therefore, considering the dose-dependent efficacy of the ERT [8, 9] and the recent real-world data reporting a disease progression after switching form agalsidase beta to migalastat [18], a switch to higher doses ERT should be considered in adult Fabry patients (aged 18–60 years) with eGFR of 45–90 ml/min/1.73 m^2^ [29], in which a linear negative slope of eGFR of 3 ml/min/1.73 m^2^/year for males (2.5 ml/min/1.73 m^2^/year for females) is observed despite stable treatment for at least 1 year [27].

On these bases, we switched to agalsidase beta Fabry patients in whom the primary therapy with agalsidase alfa or migalastat was ineffective on renal disease progression. The objective of this study was to analyse within-patient clinical outcomes, first renal measures, among those who switched their primary Fabry therapy from agalsidase alfa or migalastat to agalsidase beta in real life for a linear negative slope of eGFR of 3 ml/min/1.73 m^2^/year for males (2.5 ml/min/1.73 m^2^/year for females).

## MATERIALS AND METHODS

### Study population

This single-centre observational study was conducted at the Fabry Clinic of the University Hospital Federico II of Naples, Italy, between December 2022 and January 2025.

The included participants were adult patients (≥18 years of age) with genetically determined FD, of both genders, with eGFR between 45 and 90 mL/min/1.73 m^2^, who had switched from at least 1 year of stable treatment with the regular dose of agalsidase alfa or migalastat as their primary therapy, to agalsidase beta without any interval, for renal ineffectiveness (linear negative slope of eGFR of 3 ml/min/1.73 m^2^/year for males or 2.5 ml/min/1.73 m^2^/year for females). Participants with key exclusion criteria for ERT switch, including factors potentially able to modify renal disease progression independently from the treatment efficacy (such as recent initiation or dose change of ACE inhibitors or ARBs; Supplementary material), who changed Fabry therapy more often than twice, and with ≤90% completeness of the mandatory predefined organ and symptomatic assessment, were excluded.

Patients who fulfilled the inclusion criteria gave written informed consent and were followed-up for 24 months; those who did not complete the 24-month follow-up period were excluded from the analysis. The study was conducted in accordance with the Declaration of Helsinki and was approved by the institutional ethics committee.

### Study procedures

In patients identified by the inclusion criteria, data were retrospectively measured during the pre-switch period, from the date of the primary Fabry therapy initiation (T0) until the date of agalsidase beta initiation (switch date, T1) and prospectively during the post-switch period, from the date of switch until the end of the 24-month follow-up period (T2).

A complete clinical assessment was performed in all patients at each visit, including medical history and cardiac, renal, and neurologic evaluation. Use of concomitant medications, including pain medications and antihypertensive agents, was recorded. Patients were classified as having classic, late-onset, or other/unclassified/missing FD phenotypes using the International Fabry Disease Genotype–Phenotype database35 (version 15 August 2018).

### Study endpoints

The primary endpoint was the effect of therapy switch on renal function, evaluated by changes in eGFR slope quantified using the CKD–Epidemiology Collaboration Equation, in the two pre- and post-switch periods [11]. The estimated eGFR slopes were calculated over at least three evaluation periods using mixed-effects models.

Secondary endpoints were changes in following parameters after the switch, from pre-switch last visit (T1) to post-switch last visit (T2): clinical events including death, cardiac events (symptomatic arrhythmia requiring implantation of an implantable cardioverter-defibrillator or pacemaker, myocardial infarction, coronary artery bypass graft, or percutaneous transluminal coronary angioplasty), renal events (progression of CKD to stage 5 necessitating kidney transplantation or dialysis), and cerebrovascular events (stroke or transient ischaemic attack); 24-hour proteinuria; cardiac changes, including echocardiographic data as left ventricular mass index (LVMI) [30]; neurological changes [31, 32], determined on the basis of clinical examination, interview regarding stroke or stroke-like symptoms, and evidence from magnetic resonance imaging (MRI); changes in FD-related symptoms, including gastrointestinal pain, diarrhoea, hypohidrosis or anhidrosis, tinnitus, acroparaesthesia, pain, and fatigue; and Fabry biomarkers, including changes in lyso-Gb3 plasma concentrations.

Finally, the adverse effects (AE) considered were infusion related reactions (IARs), dyspnoea, hypertension, gastrointestinal symptoms, rigours, temperature change sensation, fever, headache, rhinitis, flushing, and pruritus.

### Statistical analysis

Data are presented as mean and standard deviation (SD) unless specified otherwise. We tested the normal distribution of our data through the Shapiro–Wilk test. Data were analysed by analysis of variance and *post hoc* analysis was performed with the Tukey test. All the analyses were performed using R Studio version 3.6.1 (R Core Team, 2019. https://www.R-project.org/). Trajectories for continuous outcomes were estimated using linear mixed models, with slopes representing the estimated annual change in the outcome during the pre- and post-switch time periods. *P* values were generated to assess the difference from 0 of the pre- and post-switch slopes and to assess the difference between pre- and post-switch slopes (*P*_pre_ _vs. post_); *P*_pre_ _vs. post_ < .05 indicates a significant difference in outcome trajectories in the two periods.

## RESULTS

### Demographic and baseline characteristics

Table 1 summarizes baseline demographics and clinical characteristics. Eleven patients were enrolled, nine men (82%) and two women (18%), who had received a primary Fabry therapy with agalsidase alfa (eight patients, 73%; six males and two females) migalastat (three patients, 17%; all males) for a mean period of 55.3 ± 31.2 months. Mean age at switch was 48.1 ± 11.0 years. A non-classic (late-onset) GLA variant (c.352C>T, p.R118W) was present in two patients (18%; one male and one female), and nine patients (82%, eight males and one female) had variants associated with the classical phenotype (c.1066C>T, p.R356W; c.950T>C, p.V317A; c.667T>G, p.C223E; and c.901C>G, p.R301G).

**Table 1:** Demographic and baseline characteristics of study population.

	Switched patients (*N* = 11)
Male, *n* (%)	9 (82%)
Age at diagnosis (years)	41.9 ± 12.6
Phenotype (classic/late-onset), *n* (%)	9/2 (82/18%)
Age at first therapy (years)	42.4 ± 12.6
Age at switch (years)	48.1 ± 11.0
Duration of pre-switch period (months)	55.3 ± 31.2
First therapy (agalsidase alfa/migalastat), *n* (%)	8/3 (73/27%)

Data are expressed as mean ± SD.

### Endpoints

In Table 2 are the results of the comparison between the parameters collected at primary Fabry therapy initiation (T0), last pre-switch (T1), and last post-switch visit (T2).

**Table 2:** Comparison between parameters collected at start of primary Fabry therapy, last pre-switch, and last post-switch visit.

Parameter	Start of primary Fabry therapy (T0)	Last pre-switch visit (T1)	Last post-switch visit (T2)
eGFR (ml/min/1.73 m^2^)	95.5 ± 16.4	67.9 ± 20.0*	67.0 ± 23.6*
Clinical events, *n* (%)	0 (0)	0 (0)	0 (0)
Proteinuria (mg/day)	559.1 ± 479.1	645.0 ± 1064.4	513.6 ± 846.6
LVMI (g/m^2^)	44.3 ± 12.2	42.5 ± 12.2	40.3 ± 11.4
Neurologic changes, *n* (%)	0 (0)	1 (9)	0 (0)
FD-related symptoms, *n* (%)	10 (91)	4 (36)*	2 (18)*
Adverse events, *n* (%)	Not applicable	2 (18)	2 (18)
Lyso-Gb3 (ng/ml)	20.5 ± 17.9	8.7 ± 8.1	6.9 ± 5.3*

Data are expressed as mean ± SD.

*Significantly different vs T0 (*P* < .05).

Regarding clinical events, no patient died, and no patient reported renal, cardiac, or cerebrovascular events during both the pre- and post-switch periods.

**Figure 1:**
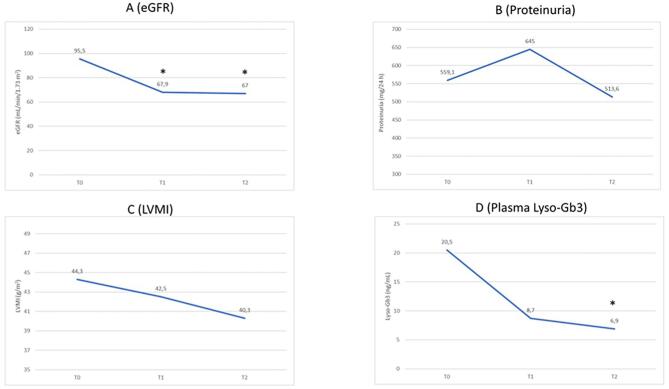
Pre- and post-switch outcome trajectories. *Significantly different vs T0 (*P* < .05).

eGFR decreased significantly from baseline to the last visit of pre-switch period (T1) (*P* < .01), while remained stable during the 24-month post-switch period, without further worsening over time (*P* = .994) (Fig. 1a). Compared to baseline, proteinuria remained stable over both treatment periods without significant change (Fig. 1b). Similarly, a slight but not significant improvement was observed in LVMI from baseline to T1 and T2, without a significant difference between the two pre- and post-switch periods (Fig. 1c). The predefined measures for neurologic function were stable over time, with one only patient reporting changes at brain MRI during the pre-switch period. Most FD-related symptoms ameliorated after starting the primary Fabry therapy (*P* < .05), and remained stable after the switch. In particular, of the 10 patients with symptoms at baseline, six reported resolution with the primary Fabry therapy and two more patients during the agalsidase beta therapy period. Plasma lyso-Gb3 levels showed a progressive reduction (although not significant) during the primary Fabry therapy (p T1 vs T0 = 0.052); the decrease continued after the switch and became significantly different from the baseline at T2 (*P* < .05), although plasma lyso-Gb3 levels did not significantly differ between the two treatment periods (*P* = .93) (Fig. 1d). It was not possible to conduct an analysis to determine potential differences between patients receiving agalsidase alfa or migalastat as primary FD therapy due to the limited sample size.

All therapies were generally safe and well tolerated, and the frequency of AE during the two treatment periods was comparable (two patients, 18% for both).

### Linear mixed models of pre- and post-switch trajectories (estimated annual change)

In Table 3 are the pre- and post-switch trajectories of most outcomes.

**Table 3:** Pre- and post-switch outcome slopes.

Parameter	Slope pre-switch	*P* value	Slope post-switch	*P* value	*P*_pre vs post_
eGFR (ml/min/1.73 m^2^)	−4.609	.00 319	−0.4545	.923	.002
Proteinuria (mg/24 h)	118.5	.0467	−65.68	.7566	.355
LVMI (g/m^2^)	−0.8557	.342	−1.141	.654	.915
Lyso-Gb3 (ng/ml)	−1.286	.2338	−0.9045	.469	.877

eGFR slope varied significantly after the switch to agalsidase beta: eGFR declined significantly during the pre-switch period (−4.61 ml/min/1.73 m^2^/year; *P* = .003), followed by stability in the post-switch period (−0.45 ml/min/1.73 m^2^/year; *P* = .923), with a significant difference between pre- vs post-switch slope (*P*_pre_ vs _post_ = .002). Proteinuria slope showed a (borderline) significant increase during the pre-switch period (118.5 mg/24 h/year; *P* = .047), whereas it remained stable after the switch (−65.68 mg/24 h/year; *P* = .757), although the difference between the slopes of the two periods was not statistically significant. LVMI and plasma lyso-Gb3 trajectories showed stability over both treatment periods (LVMI pre-switch −0.856 g/m^2^/year, *P* = .342, post-switch −1.1 g/m^2^/year, *P* = .654; Lyso-Gb3 pre-switch −1.29 ng/ml/year, *P* = .234, post-switch −0.904 ng/ml/year, *P* = .469) , without significant differences between the slopes of pre- and post-switch period.

## DISCUSSION

It has been recently suggested that a treatment for classic FD should be considered effective in preserving renal function when it maintains an eGFR decline <1 ml/min/1.73 m² per year [27]; conversely, a therapy should be deemed ineffective when the eGFR loss remains ≥3 ml/min/1.73 m² per year (≥2.5 ml/min/1.73 m² per year in females), thereby identifying patients at risk of disease progression who may benefit from a treatment switch [27]. Clinical guidelines for FD management also recommend switching treatment when individualized therapeutic goals are not met [28].

For a long time, there has been debate regarding the most effective therapy for FD. In particular, the two ERT formulations are biochemically very similar and have both demonstrated safety and efficacy in FD [5, 33, 34], but have different approved dosages, with agalsidase beta being administered at a 5-fold higher dose compared to agalsidase alfa [6, 7]. Since recent studies highlighted a dose-dependent effect of agalsidase, showing that higher doses enhance intracellular enzymatic activity, improving Gb3 clearance [8, 9] and slowing nephropathy progression [10, 11], agalsidase beta received full FDA approval in 2021 [35] and has been recently recommended as first-line therapy, particularly in classic male Fabry patients [36]. Regarding migalastat, although clinical trials and registry data support its efficacy and tolerability [14–16], real-world data predominantly indicate that patients who switched from previous ERT to migalastat experience a more pronounced decline in eGFR [17–19].

Considering the dose-dependent efficacy of ERT [8, 9] and reports of disease progression following a switch from agalsidase beta to migalastat [18, 37, 38], we switched patients to agalsidase beta who, despite ongoing treatment with agalsidase alfa or migalastat, exhibited a rapid progression of renal damage, meeting the criteria for ineffective renal response to therapy.

The present study aimed to evaluate renal and clinical outcomes in Fabry patients switched from agalsidase alfa or migalastat to agalsidase beta due to insufficient renal efficacy. Main results align with existing literature, demonstrating that switching to agalsidase beta resulted in improved renal function and reduced lyso-Gb3 levels. Specifically, the significant decline in eGFR observed during the pre-switch period stabilized post-switch. In patients in whom the primary Fabry therapies were ineffective in preserving renal function, as evidenced by an eGFR slope of −4.61 ± 3.4 ml/min/1.73 m²/year, agalsidase beta significantly mitigated eGFR decline, reducing the slope to −0.45 ± 2.4 ml/min/1.73 m²/year. Moreover, during the initial period of primary Fabry therapy, proteinuria exhibited a slight, borderline significant, worsening trend. However, after the switch to agalsidase beta, proteinuria remained stable over time, suggesting a potential benefit of the higher-dose ERT in Fabry nephropathy. In accordance with the exclusion criteria, no patient received any pharmacological agents known to influence renal disease progression or proteinuria independently of Fabry-specific therapy throughout the follow-up period (Supplementary Material). Regarding plasma lyso-Gb3 levels, a progressive yet non-significant reduction was noted during the primary Fabry therapy period (*P* T1 vs T0 = .052). However, following the switch to agalsidase beta, the reduction reached statistical significance (*P* < .05), reinforcing the evidence of the dose-dependent effect of ERT on lyso-Gb3 clearance [8, 9]. Furthermore, the incidence of clinical events, proteinuria, and cardiac and neurological functions remained stable across both treatment periods, and the therapy switch was well tolerated.

Several limitations should be considered. First, the small sample size limits statistical power and generalizability. Second, the study’s ‘switch’ design, while mitigating potential confounders, introduces age-related biases, as patients were older during the post-switch period. Given that eGFR and certain cardiac measures naturally deteriorate with age, this may confound the interpretation of last pre-switch vs. last post-switch assessments. However, differences in slope trajectories between pre- and post-switch periods suggest a genuine therapeutic effect rather than an age-related trend. Furthermore, pre-switch and post-switch comparisons relied on single assessments per treatment period, increasing the likelihood of measurement variability. Finally, it was not possible to conduct an analysis to determine potential differences between patients receiving agalsidase alfa or migalastat as primary FD therapy, due to the limited sample size. Similarly, we could not evaluate differences between patients with classical and late-onset variants for the same reason.

Despite these limitations, the study offers valuable insights. The within-patient comparison design eliminates inter-patient variability, thereby enhancing the reliability of our findings. By analysing clinical outcomes in the same individuals before and after the switch, we minimized confounding factors that might arise in studies comparing separate patient cohorts. Furthermore, our study provides real-world evidence on the effectiveness of treatment switching, complementing controlled clinical trials with data from routine clinical practice. An additional limitation may be the unequal duration of the observation periods before and after the therapy switch (i.e. T0 to T1 versus T1 to T2), which could have influenced the comparability of the eGFR slopes. Furthermore, the more pronounced renal decline before the switch may have prompted intensified clinical monitoring and supportive interventions (e.g. dietary counselling, more frequent visits) during the post-switch period, potentially contributing to the stabilization of renal function.

In conclusion, our findings underscore the benefits of switching to agalsidase beta in patients experiencing progressive renal decline under agalsidase alfa or migalastat therapy. These results strengthen the growing body of evidence supporting for dose optimization in ERT to attenuate FD progression and improve renal outcomes. Moreover, our findings confirm that agalsidase beta allows for better control of renal function and disease progression, as demonstrated by its impact on eGFR slope stabilization and significant reduction in lyso-Gb3 levels. Future studies with larger sample sizes and longer follow-up durations are necessary to further validate these findings.

## Supplementary Material

sfaf318_Supplemental_File

## Data Availability

The data underlying this article will be shared on reasonable request to the corresponding author.
