# Supplementary material for: Clinical outcomes in Fabry patients switching to agalsidase beta for renal ineffectiveness of the primary Fabry therapy: a single-centre analysis
Source: Clin Kidney J. 2025 Oct 15;18(11):sfaf318. doi: 10.1093/ckj/sfaf318 (PMC12686814; doi:10.1093/ckj/sfaf318)
Supplement: sfaf318_Supplemental_File [file sfaf318_Supplemental_File.docx]

**Supplementary material.** Key exclusion criteria for ERT switch.

1. History of anaphylaxis or Type 1 hypersensitivity reaction to agalsidase beta

2. Known non-pathogenic Fabry mutations

3. History of renal dialysis or transplantation

4. History of acute kidney injury in the last 12 months prior to evaluation, including specific kidney diseases (e.g., acute interstitial nephritis, acute glomerular and vasculitis renal diseases); non-specific conditions (e.g., ischemia, toxic injury); as well as extrarenal pathology (e.g., prerenal azotaemia, and acute postrenal obstructive nephropathy)

5. Patient with a screening eGFR value between 91-120 mL/min/1.73 m^2^, having an historical eGFR value higher than 120 mL/min/1.73 m^2^ (during 9 to 18 months before evaluation)

6. Angiotensin converting enzyme (ACE) inhibitor, angiotensin receptor blocker (ARB) or Sodium Glucose Cotransporters 1 (SGLT1) inhibitors therapy initiated or dose changed in the 4 weeks prior to evaluation

8. Cardiovascular event (myocardial infarction, unstable angina) in the 6-month period before evaluation

9. Congestive heart failure NYHA Class IV

10. Cerebrovascular event (stroke, transient ischemic attack) in the 6-month period before evaluation

12. Female subjects who are pregnant, planning to become pregnant during the study, or are breastfeeding

13. Presence of any medical, emotional, behavioural or psychological condition that, in the judgment of the Investigator and/or Medical Director, would interfere with the patient’s compliance
